# Treatment of Comorbid Alcohol Dependence and Anxiety Disorder: Review of the Scientific Evidence and Recommendations for Treatment

**DOI:** 10.3389/fpsyt.2017.00173

**Published:** 2017-09-22

**Authors:** Carmen Gimeno, Marisa Luisa Dorado, Carlos Roncero, Nestor Szerman, Pablo Vega, Vicent Balanzá-Martínez, F. Javier Alvarez

**Affiliations:** ^1^Unit for Addictive Behaviours, Conselleria de Sanitat, Alicante, Spain; ^2^Sociedad Española de Patología Dual, Madrid, Spain; ^3^Unit for Addictive Behaviors of Guillen de Castro, Conselleria de Sanitat, Valencia, Spain; ^4^Addiction and Dual Diagnosis Unit, Psychiatric Service, Hospital Vall Hebron-ASPB, CIBERSAM, Psychiatric Department, Universitat Autònoma de Barcelona, Barcelona, Spain; ^5^Salud Mental Retiro, Hospital General Universitario Gregorio Marañon, Madrid, Spain; ^6^Instituto de Adicciones, Madrid, Spain; ^7^Service of Psychiatry, La Fe University and Polytechnic Hospital, Department of Medicine, Medical School, University of Valencia, CIBERSAM, International Society for Nutritional Psychiatry Research (ISNPR), Valencia, Spain; ^8^Pharmacology, Faculty of Medicine, University of Valladolid, Valladolid, Spain; ^9^CEIC/CEIm, Hospital Clínico Universitario de Valladolid, Valladolid, Spain

**Keywords:** alcohol-use disorder, anxiety disorders, comorbidity, co-occurring disorders, treatment recommendations

## Abstract

Patients with alcohol-use disorders (AUDs) have a high prevalence of anxiety disorders (AnxDs). “Co-occurring disorders” refers to the coexistence of an AUD and/or drug related disorders with another non-addictive psychiatric disorder. The aim of this study was to assess the effectiveness of psychopharmacological treatments and psychotherapy in patients with AUD and AnxD and to propose recommendations for the treatment of patients with comorbid AnxDs and AUDs. Randomized clinical trials, meta-analyses, and clinical guidelines were retrieved from PubMed, Embase, and Cochrane databases. Paroxetine was found to be effective in social anxiety patients with alcohol dependence. Selective serotonin reuptake inhibitors (SSRIs), especially sertraline, showed effective results in posttraumatic stress disorder and in comorbid AnxD–AUD. However, SSRIs should be used with caution when patients are actively drinking because they may increase alcohol consumption. Buspirone, gabapentin, and pregabalin were found to be effective in comorbid AnxD–AUD. The treatment of dual AnxDs should start as early as possible. Since AUDs and AnxDs can reinforce each other, treatments targeting both pathologies can be effective. Women suffer from higher levels of stress and AnxDs than men, and they are also more vulnerable to maintaining alcohol consumption levels. Further research is needed in this comorbid patient population, including the study of different types of patients and gender perspectives.

## Introduction

Anxiety disorders (AnxDs) are often combined with alcohol-use disorders (AUDs), worsening the symptoms, and making treatment more difficult. Comorbid AnxDs and AUDs are associated with poorer treatment results and increased difficulties in treatment with standard psychosocial interventions (1). Problematic alcohol consumption is defined as a pattern that causes mental and physical health damage and serious alterations in the subject’s life and in the lives of those in his or her social environment and represents a major political concern (2, 3).

Anxiety disorders are characterized by the presence of fear, excessive anxiety, and behavioral changes. Fear is an emotional response to an immediate threat, real or imagined, while anxiety is an anticipatory response to a future threat, real or imagined. Fear is typically associated with the autonomic activation needed for defense and flight, and fearful thoughts (imminent danger) are associated with flight conduct. Anxiety is associated with muscular tension, awareness of future danger, and cautious and avoidant behavior (2). Posttraumatic stress disorder (PTSD) has also been included, following the American Psychiatric Association Diagnostic Classification Diagnostic and Statistical Manual of Mental Disorders (DSM)-IV-TR (4).

In general, the terms “dual disorders” or “co-occurring disorders” are used when referring to the broad clinical patient population suffering from substance use and other psychiatric disorders.

According to the National Institute on Drug Abuse (NIDA), the term “comorbidity” describes two or more disorders or illnesses occurring in the same person. These disorders can occur simultaneously or consecutively. Comorbidity also implies interactions between the illnesses that can worsen the course of both (5).

The European Monitoring Center for Drugs and Drug Addiction defines “comorbidity/dual diagnosis” as the “temporal coexistence of two or more psychiatric disorders as defined by the International Classification of Diseases” (6).

The comorbidity of alcohol dependence with another psychiatric disorder is very common (7–10), while the association of AUDs with other neuropsychiatric illnesses, such as depression or AnxDs, is also very frequent (11–14).

The diagnosis is challenging due to the possibility of multiple causes. Symptoms of anxiety may be caused by abstinence or intoxication effects (induced AnxDs). It is also possible that one disorder may be, directly or indirectly, induced by the other. For example, a subject may turn to substance consumption as a means to cope with anxiety; AnxDs may be triggered by the stress associated with substance consumption (15). The observation of anxiety symptoms in patients who have been abstinent for several weeks allows us to determine whether the patient is presenting with abstinence- or intoxication-induced anxiety or whether they should be diagnosed with primary AnxD (16).

There are various theoretical models defining the relationship between AUDs and AnxDs. The models that give priority to AnxDs generally accept the idea that it is the effort of coping with situations that provokes anxiety, which in turn leads to alcohol abuse. These models include the tension reduction hypothesis (17) and the self-medication hypothesis (18), and they can be particularly relevant in cases of AnxD that normally precede the emergence of dependency, such as general AnxD and agoraphobia (17–19). In fact, the evidence of a causal relationship between AnxD symptoms and the symptoms of AUDs includes a possible dose–response relationship between the severity of the symptoms of social phobia and the level of alcohol consumption (20), as well as the finding that early treatment of AnxDs reduces alcohol abuse (1, 21, 22).

Alcohol is a psychotropic depressant of the central nervous system that promotes simultaneous changes in several neuronal pathways, exerting a profound neurological impact that leads to various behavioral and biological alterations (23).

The direct effect of alcohol on several neurotransmitter receptors [gamma-aminobutyric acid [GABA], glutamate, endocannabinoids AEA and 2-AG, among others] has been described (23). More specifically, alcohol enhances the GABA action and antagonizes glutamate action (24). On the other hand, GABAergic and glutamatergic systems seem to be involved in AnxD, such as Generalized anxiety disorder (GAD), panic disorder, and social AnxD (25). Furthermore, glutamate signaling mediates certain aspects of the intoxicating and rewarding effects of alcohol and chronic alcohol abuse produces a hyperglutamatergic state (26).

One of the five major components of the glutamate system, the *N*-methyl-d-aspartate (NMDA) receptor (27), could play a role in the risk of benzodiazepine abuse. In this sense, the NMDA receptor antagonist-benzodiazepine has been involved in abuse liability (28). Thus, this medication should be used very carefully when treating AUD patients.

However, different classifications of alcohol users could be considered. Cloninger et al. (28) defined two types of alcoholism, based on alcohol-related symptoms, personality traits, age of onset, and patterns of inheritance. Type 1 alcoholism was defined by anxious personality traits and the rapid development of tolerance and dependence on the antianxiety effects of alcohol. Babor et al. (29) also focused on two types: Type A was characterized by a later onset, fewer childhood risk factors, less severe dependence, fewer alcohol-related problems, and less psychopathological dysfunction. This typology, which is similar to Cloninger’s types, could help explain the different causes, courses, prognoses, and outcomes for the disorder (30).

It is estimated that more than 51% of people with psychiatric disorders abuse or depend on psychoactive substances (11, 14), especially alcohol. The presence of a psychiatric disorder increases the risk of progressing from mere consumption to alcohol dependency (31). In fact, 37% of AUD patients suffer from other psychiatric disorders. Alcohol dependence is associated with an increased risk of mood disorders (three times higher), AnxDs in general (twice as high), generalized AnxD (four times higher), panic disorders (almost double), and posttraumatic stress disorder (twice as high) (32–34).

Anxiety disorders and other psychiatric disorders in patients with substance-use disorders (SUDs) have a significant impact on treatment results (35, 36). Anxiety, considered as a symptom, decreases in abstaining patients, yet if it remains present, the risk of a relapse is two times higher (37–40).

“Off-label prescription” indicates that the medication is being used in a manner not specified in the approved Summary of Product Characteristics (therapeutic indications in other conditions, unapproved changes in dosage and/or duration of treatment) (41). However, such situations, which should be exceptional, are frequent in clinical practice, especially in a field such as psychiatry, and they occur frequently in patients with comorbidity (42) whose situations require drug combinations (43). The Agency for Healthcare Research and Quality (44) stated that the most common uses of psychiatric drugs unapproved on the labeling appear in the treatment of depression, obsessive–compulsive disorder (OCD), PTSD, personality disorders, Tourette syndrome, autism, and agitation in dementia (44).

Currently, there are many drugs approved for the isolated treatment of AnxDs or AUDs. However, there is little certainty in cases of patients with comorbid AnxDs and AUDs, and it is not clear whether the recommendations issued separately for patients with AnxDs or AUDs can be accepted in comorbid patients. Furthermore, several studies have been carried out using non-approved medicines (off-label prescription) in patients with AUD and AnxD.

The aim of this study was to assess the effectiveness of psychopharmacological treatments and psychotherapy in patients with AUD and AnxD, and to propose recommendations for the treatment of patients with comorbid AnxDs and AUDs. The influence of age and gender were also analyzed.

## Materials and Methods

### Literature Search

The authors undertook an extensive review of publications, meta-analyses and Randomized Clinical Trials (RCTs), addressing AnxD and AUD treatments. Database searching through July 2016, was carried out using MEDLINE/PubMed, Embase, and Cochrane Database of Systematic Reviews platforms.

In a first stage, a search was performed using the terms: (i) “anxiety disorders” and “anxiety symptoms”; (ii) “alcohol addict,” “alcohol abuse” and “alcohol,” (iii) “naltrexone,” “disulfiram,” “atypical antipsychotics,” “antidepressants,” “SSIRs,” “mood stabilizer,” “anticonvulsants,” “buspirone,” and “benzodiazepine.”

In a second stage, a search combining the results obtained in the previous searches was performed, aiming at identified articles that contained at least one term from each of the three identified groups (anxiety, alcohol, and psychotropic drugs).

A total of 121 works were identified, of which 50 were RCTs. After a thorough manual selection process, the total was reduced to 21, having discarded publications that did not fulfill the RCT criteria or did not cover AnxD and AUD. No meta-analysis was found on the topic.

### Clinical Guidelines Analysis

The main clinical guidelines were reviewed in a parallel analysis to contrast the evidence and degrees of scientific recommendations. The search strategy was applied based on the following terms.

- In MEDLINE/PubMed: using the following key words “guidelines of alcohol abuse,” “harmful use,” “addiction and comorbidity addiction,” and “dual diagnosis.”
- In the RIMA Network (https://www.rima.org): searching for guidelines through the psychiatric portal.

Guidelines of the British Association of Psychopharmacology (BAP) were obtained (45).

Moreover, scientific associations’ Web pages were consulted for works on scientific evidence in this field of knowledge, and the names and “URLs” are mentioned in Table 1.

**Table 1:** Scientific institutions and associations web pages consulted for clinical guidelines on treatment of comorbid AnxD–alcohol-use disorders patients.

British Association of Psychopharmacology Guidelines	http://www.bap.org.uk/docsbycategory.php?docCatID=2
National Institute on Drug Abuse (NIDA)	http://www.drugabuse.gov/publications/research-reports/comorbidity-addiction-other-mental-illnesses
Agency for Healthcare Research and Quality	http://www.guideline.gov/browse/by-topic-detail.aspx?id=38655&ct=1
National Clearinghouse Guideline	
National Institute of Health and Care Excellence (NICE)	http://www.nice.org.uk/
American Psychiatric Association	https://www.appi.org/
Canadian Network for Mood and Anxiety Treatments	http://www.canmat.org/
Addiction Treatment Forum	http://atforum.com/addiction-resources/evidence-based-resources-for-addiction-professionals/
Evidence-based practice for substance-use disorders	http://adai.uw.edu/ebp/ebpresources.htm
Substance Abuse and Mental Health Service Administration	http://www.samhsa.gov/ebpwebguide/appendixB_Treatment.asp
Science Direct	http://www.sciencedirect.com/science/book/9780123743480
Samhsa/csat Treatment Improvement Protocols	http://www.ncbi.nlm.nih.gov/books/NBK82999/
Socidrogalcohol, Guías Clínicas Basadas en la Evidencia Científica, 3° ed.	http://www.socidrogalcohol.org/

### Level of Evidence

We used the recommendations suggested by the Center of Evidence-Based Medicine of Oxford (46), to establish levels of evidence and make recommendations based on the RCT identified.

## Results

Below is a description of the most relevant findings, based on the content analysis of comorbid AUD–AnxD treatment (Table 2).

**Table 2:** Pharmacological treatments of comorbid AnxDs and AUDs patients: main scientific publications and findings.

Author/team	Clinical condition	Trial design/duration	Sample/doses	Results
**Atypical antipsychotics: quetiapine**				

Monnelly et al. (61)	PTSD	Retrospective Controlled/12 months	*N* = 50/25–200 mg/night	The average abstinence duration was significantly longer. Hospitalization was shorter in the quetiapine group; the difference was statistically significant

**Antidepressants NaSSAs: mirtazapine**				

Liappas et al. (64)	Social anxiety	Open/4–5 weeks after detoxification period	*N* = 54/30–60 mg/daily	There was high remission probability in social anxiety symptoms in AUD in-patients after alcohol detoxification. The study provided preliminary evidence that psychotherapeutic treatment combined with mirtazapine (30–60 mg/day) had a greater impact on symptoms than pharmacological treatment alone.A longer follow-up period was needed to determine remission levels

**Non-selective monoamine reuptake inhibitors antidepressants: nefazodone + psychotherapy**				

Hernadez-Avila et al. (67)	Anxiety symptoms in AUD patients with major depression	RCT/10 weeks	*N* = 41/200–600 mg/day	Depression and anxiety symptoms decreased significantly with time. The nefazodone group showed higher symptom reduction; however, the difference did not attain statistical significance. Nefazodone patients showed significantly greater symptom reduction and a reduction in the total number of drinks on days of excessive drinking, compared with subjects treated with placebo. The lack of significant effects on depression and anxiety symptoms might reflect limitations of the statistics. Despite the rather small size of the sample group, nefazodone helped to reduce some means of consumption in the depressive AUD patients

**Trazodone**				

Janiri et al. (65)	Anxiety	Open/12 weeks	*N* = 25/50–100 mg/day	In the detoxified AUD patient sample, trazodone showed anxiolytic, anti-depressive, and anti-craving efficacy. Little evidence, small sample, open study

**Paroxetine**				

Randall et al. (69)	Social anxiety	RCT/8 weeks	*N* = 15/20–60 mg/day	Paroxetine was effective in social anxiety and AUD. Patients with alcohol dependence—6 with social anxiety who were given paroxetine and 9 who were given a placebo over 8 weeks—were compared. The paroxetine group improved significantly in one of the social anxiety indicators (effect size = 0.81)

Thomas et al. (70)	Social anxiety	RCT/16 weeks	*N* = 42/40–60 mg/day	Paroxetine reduced social anxiety and alcohol consumption (self-medication use for social phobia)

Book et al. (71)	Social anxiety	RCT/16 weeks	*N* = 42/40–60 mg/day	Paroxetine reduced social anxiety

**Sertraline**				

Brady et al. (73)	PTSD	RCT/12 weeks	*N* = 94/150 mg/day	Sertraline was associated with better mood and drinking outcomes in the subgroup with early-onset PTSD and later onset and less severe alcohol dependence, whereas among those with later-onset PTSD and more severe alcohol problems, sertraline was not better than the placebo. There could be subtypes in patients with severe or moderate AUD and early or later-onset PTSD that require different therapeutic strategies

Pettinati et al. (75)	Alcohol-dependent subjects	RCT/14 weeks	*N* = 100/200 mg/day	A significant interaction between the alcoholic subtype and the medication condition was found, confirming the findings. Alcoholic subtypes responded differentially to serotonergic medications. Somewhat at variance with their results, however, the present study showed that the lower risk/severity (Type A) subjects had more favorable outcomes when treated with sertraline, compared to the placebo

Labbate et al. (76)	PTSD	RCT/12 weeks	*N* = 93/50 mg/day	Anxiety or mood disorder comorbidity did not decrease treatment response in individuals with comorbid PTSD and an alcohol-use disorder

**Anxiolytics: buspirone**				

Kranzler et al. (77)	Generalized anxiety	RCT/12 weeks	*N* = 61/Maximum dosage of 60 mg/day	Buspirone therapy was associated with greater retention in the 12-week treatment trial, reduced anxiety, a slower return to heavy alcohol consumption, and fewer drinking days during the follow-up period. GAD and AUD patients received weekly relapse prevention psychotherapy

Bruno (81)	Anxiety	RCT/8 weeks	*N* = 50/15–30 mg/day	Buspirone significantly reduced the Hamilton Anxiety Rating Scale total scores (*p* = 0.006)

Tollerfson et al. (82)	Social anxiety	RCT/24 weeks	*N* = 51/60 mg/day	Buspirone was superior to placebo as an anxiolytic

**Gabapentin**				

Myrick et al. (85)	Anxiety	RCT/12 days	*N* = 84/900–120 mg/day	Participants treated with a high (1,200 mg) or a low (900 mg) dose of gabapentin had lower probabilities of drinking during the treatment period and during a 1-week follow-up posttreatment period. During the trial period, the subjects treated with the high dose of gabapentin had lower anxiety levels and a better capacity to perform tasks than the subjects treated with lorazepam. The gabapentin groups also had less craving, anxiety, and daytime sedation, compared to lorazepam. During treatment, low-dose gabapentin-treated participants had less craving and anxiety, as well as less depression syndromes

**Pregabalin**				

Di Nicola et al. (87)	AUD	Open, Prospective/14 days	*N* = 40/200–450 mg/day	Alcohol withdrawal symptoms and craving for alcohol were significantly reduced over time after pregabalin treatment. Pregabalin also resulted in a favorable improvement in psychiatric symptoms and quality of life

Martinotti et al. (88)	AUD and anxiety (SCL-90)	RCT/pregabalin/naltrexone/16 weeks	*N* = 71/150–450 mg/day pregabalin; 50 mg/day naltrexone	Compared with naltrexone, pregabalin resulted in a greater improvement in specific symptoms in the areas of anxiety, hostility, and psychosis and the duration of abstinence from alcohol. Pregabalin also resulted in a better outcome in patients reporting a comorbid psychiatric disorder. Results from this study globally placed pregabalin within the same range of efficacy as naltrexone

Martinotti et al. (89)	AWS	RTC, pregabalin/lorazepam/tiapride/14 days	*N* = 111/maximum doses: pregabalin 450 mg/day; tiapride 800 mg/day; lorazepam 10 mg/day	Pregabaline, lorazepam, and tiapride showed evidence of safety and efficacy in the treatment of uncomplicated forms of AWS. The efficacy of pregabalin was superior to that of tiapride, and, for some measures, to that of, lorazepam

**Opioid antagonist: naltrexone**				

Petrakis et al. (92)	Anxiety	RCT/12 weeks	*N* = 254/50 mg/day Naltrexone; 250 mg/day disulfiram	There was a high rate of abstinence across groups. Subjects treated with an active medication had significantly more consecutive weeks of abstinence and less craving than those treated with a placebo, but there were no significant group differences in other measurements of alcohol consumption. There was no advantage in the combination of both medications

Petrakis et al. (93)	PTSD	RCT/12 weeks	*N* = 254/50 mg/day Naltrexone; 250 mg/day disulfiram	Subjects with PTSD had better alcohol outcomes with active medication (naltrexone, disulfiram, or the combination) than they did on a placebo; the overall psychiatric symptoms of PTSD improved. Individuals with PTSD were more likely to report some side effects when treated with the combination. Curiously, the group with only disulfiram also reported fewer PTSD symptoms. This finding represents a potentially confusing factor because it seems possible that it is abstinence that might be responsible for the improvement in PTSD symptoms. It is suggested that patients whose aim in treatment is to abstain from alcohol might be more prone to experiencing a reduction in PTSD symptoms

**Psychopharmacological strategy: pharmacological treatment and psychotherapy**				

Schade et al. (95)	Social anxiety/social phobia	RCT/32 weeks	*N* = 96	Alcohol dependence and comorbid social phobia and/or agoraphobia patients were randomly assigned to an intensive psychosocial relapse prevention programme (*n* = 49) or, in combination, to an anxiety treatment programme that included CBT and optional pharmacotherapy using SSRIs. Although the combined therapy clearly reduced anxiety symptoms, it had no significant effects on alcohol relapse levels

Randall et al. (72)	Social anxiety	RCT/12 weeks and 3 months after the end of treatment	*N* = 93	The design was a two-group, randomized clinical trial using 12 weeks of individual (CBT) for alcoholism only (*n* = 44) or concurrent treatment for both alcohol and social anxiety problems (*n* = 49). The results showed that both groups improved (there was no significant difference) in alcohol-related outcomes (percentage of abstinence days and days of excessive drinking); moreover, both groups improved in social anxiety and phobia during the treatment and follow-up periods

*AnxD, anxiety disorder; AUD, alcohol-use disorder; AWS, alcohol withdrawal syndrome; CBT, cognitive behavioral therapy; GAD, generalized anxiety disorder; NaSSAs, noradrenergic and specific serotonergic antidepressants; PTSD, posttraumatic stress disorder; RCT, randomized clinical trials; SNRIs, serotonin noradrenalin reuptake inhibitors; SSRI, selective serotonin reuptake inhibitor*.

### Treatment Results—General Observations

The bibliographic analysis showed a heterogeneous picture of the combined effects of AnxDs and AUDs. There is no certainty of the impact that AnxD has on the results of alcohol consumption; although some studies suggested similar treatment results in persons with or without comorbid AnxD, a poorer prognosis was suggested by others (15).

A recent clinical trial (47) performed in primary care with 1,004 patients and an 18-month follow-up period analyzed the relationship between AnxDs and AUDs. It examined the impact of alcohol-use symptom severity (patients with alcohol abuse were included, but patients with alcohol dependence were excluded) on AnxD treatment outcomes. The majority of analyses revealed no predictive effects of alcohol-use severity on outcome; however, baseline alcohol problems were associated with somewhat higher anxiety and depression symptoms at the 18-month follow-up. It was concluded that there might be no need to postpone treatment of AnxDs until alcohol problems were addressed. Moreover, it was indicated that alcohol consumption should not modify a therapeutic approach in AnxD patients. It was concluded that delaying treatment for anxiety until the alcohol consumption problems had been resolved was not necessary, especially concerning patients who had not reached dependency criteria. Taking into account the hypothesis of consumption as self-medication, in which the patient takes to alcohol in order to calm his/her symptoms, it can be deduced that treating the anxiety will help to limit the alcohol consumption and its persistence, as well as avoiding any worsening of the consumption. The combined treatment models would include cognitive and pharmacological therapy, either singly or combined (47). Level of evidence: 1b.

### Gender Considerations

Several studies have attempted to evaluate possible gender differences in the frequency of comorbid AnxDs and AUDs. Population surveys have consistently shown that AnxDs are more common among women, whereas AUDs are more common among men (48).

Women have an increased likelihood of independent AnxDs compared with men, but prior AnxDs were also more strongly predictive of later alcohol dependence among women.

Regarding typology, gender showed some differences. For example, Cloninger’s type II was essentially limited to men, while in the Babor et al. study (29), men were equally classified as type A or type B, but women were slightly more frequently classified as type A. Therefore, men were more frequently assigned to types II or B (29).

A multicentre trial found that AnxDs had a substantial influence on the course and severity of alcoholism in women (49). The women with AnxDs had faster dependence evolution, including earlier first drink onset and shifting to regular consumption and a greater incidence of abstinence symptoms. One potential explanation is that the reasons for using alcohol can differ by gender. For example, women might be more prone than men to self-medicate with alcohol for mood problems (50–52). Furthermore, empirical inspection of gender differences in stress-related drinking has shown that women report higher levels of stress and have a stronger link between stress and drinking (53, 54).

In turn, evidence has revealed in studies performed over the last 25 years a high comorbidity of PTSD and AUDs (55, 56). Another study concluded that there was a two times greater propensity to develop alcohol dependency [odds ratio (OR) = 1.85] in women exposed to trauma, while women with PTSD exposed to trauma were even more likely (OR = 3.54) to develop alcohol dependency than women with only trauma (54, 55).

### Age Considerations

Multiple forms of anxiety psychopathology are associated with alcohol use problems in adolescents. A recent crosssectional study (21) provided preliminary evidence of two cross-diagnoses: distress tolerance (DT) and anxiety sensitivity. A crosssectional study that examined a sample of 534 high school students (14–15 years old) found that the multiple manifestations of anxiety were associated with greater alcohol use problems; however, this association was not found in the case of DT. The authors suggested that anxiety sensitivity could be an important trans-diagnostic target for alcohol prevention programs for those in early adolescence who experience elevated anxiety symptoms.

In addition to alcohol consumption disorders, child care negligence or childhood adversities were associated with a greater risk of PTSD (54). Studies in samples of individuals seeking treatment for AUDs have also found a high prevalence of reported childhood adversity and PTSD. In a study in men and women treated for SUDs, 26% reported having been victims of childhood physical or sexual abuse (14). A review of studies of individuals seeking treatment for addictions revealed rates of PTSD as high as 50% or higher (54–57). The development of PTSD usually precedes the development of SUDs.

### Comorbid AnxD and AUD Pharmacological Treatment Considerations

#### Atypical Antipsychotics

Some authors (58, 59) have found that quetiapine may be a promising agent for non-comorbid GAD, whereas more studies are needed before making practical recommendations on the use of olanzapine and risperidone. Risperidone and olanzapine add-on could play a role in resistant or chronic posttraumatic stress disorder patients, although only the addition of risperidone can be recommended on the basis of the criterion of two or more positive placebo-controlled trials (60).

However, there have been few studies on the use of antipsychotics in AnxD and AUD patients. A retrospective controlled study (61) found that the mean number of abstinence days was significantly greater and the number of hospitalizations was significantly lower for the quetiapine group during the period studied. Level of evidence: 4.

A review from an expert opinion on aripiprazole might provide a novel therapeutic approach for alcoholism by attenuating craving (during the abstinence phase) and anxiety, depression, and impulsivity, which are potentially responsible for relapses and the worsening of drinking behaviors. Subjects with a Cloninger II personality profile are characterized by higher levels of impulsiveness, sensation seeking, early onset of alcohol misuse, antisocial behavior and reward craving. In these conditions, aripiprazole has shown some level of evidence in different studies (62, 63). Level of evidence: 4 (Table 2).

#### Use of Antidepressants

There is preliminary evidence that combined psychotherapeutic/mirtazapine treatment (30–60 mg/daily) has a greater impact on AnxD symptoms than non-pharmacological treatment alone. These authors also found a high incidence of social anxiety symptoms subsiding in in-patient alcoholics following alcohol detoxification (64). Level of evidence: 4.

Trazodone is a widely used drug in clinical practice (65, 66). However, we found only one open study with 25 AUD patients. The authors reported anxiolytic, anti-depressive and anti-craving effects of trazodone when patients were treated with a dose of 50–100 mg/day (65). Level of evidence: 4.

The use of antidepressants was tested in a RCT in AUD patients with comorbid AnxD and major depression. Subjects were randomly assigned to receive nefazodone and supportive psychotherapy. Depressive and anxiety symptoms declined significantly over time. Although the nefazodone group showed greater reductions in these symptoms, the effects did not attain statistical significance. Nonetheless, nefazodone-treated subjects showed a significantly greater reduction in heavy drinking days and in total drinks compared with placebo-treated subjects (67). Level of evidence: 1a.

Several reviews have found good results with the selective serotonin reuptake inhibitors (SSRIs) in comorbid AnxD and AUD treatment (68). Paroxetine is the antidepressant for which the most scientific evidence was found for dual anxiety treatment (68–70). Three clinical trials (69–71) found that paroxetine was effective in social anxiety patients with alcohol dependency. Level of evidence: 1a.

Other authors have emphasized the importance of combinations of psychotherapeutic interventions/treatments combined with pharmacological SSRI treatment or topiramate when managing comorbid PTSD and alcohol dependency patients (71–73).

In turn, a systematic review emphasized that drugs such as SSRIs, especially sertraline, were effective in PTSD and in comorbid AnxD-AUD (74). Two randomized clinical trials (75, 76) found evidence of sertraline treatment efficacy in alcoholics with PTSD. However, although sertraline can be effective in PTSD treatment (55), there is little evidence for SSRI efficacy in alcohol consumption. Level of evidence: 1a.

Moreover, other studies have suggested that there might be alcoholic subtypes that respond differentially to serotonergic medication (75). One detailed analysis showed that sertraline treatment in patients whose traumatic experience occurred prior to the development of alcohol dependence improved drinking results. However, sertraline-treated patients with grave dependence and PTSD drank more than placebo-treated patients. Level of evidence: 1a.

A significant interaction between the alcoholic subtype and the response to medication was found in a randomized controlled trial with 100 AUD patients (75), confirming previous findings (77) that alcoholic subtypes responded differently to serotonergic medications. Pettinati’s trial showed that those with lower risk/severity of alcoholism had more favorable drinking outcomes in response to treatment with sertraline than with a placebo (75). Level of evidence: 1a.

The Socidrogalcohol clinical guidelines recommend (78) using SSRIs with caution if the patient is actively drinking because the agents can lead to an increase in alcohol consumption in some patients (79). Level of evidence: 1b.

However, these guidelines also state that SSRIs are preferred in comorbid AnxD and AUD treatment due to their low abuse potential, rare interactions and low overdose risk. The recommended dosage follows the insert indications. There was also a suggestion that panic-crisis patients with an alcohol consumption history or AnxD family history could be recommended for an early start of AnxD treatment without waiting for abstinence (78).

#### Use of Anxiolytics

The latest Good Practice Recommendations of the French Alcohol Society and the guidelines of the BAP (45, 80) suggest the use of benzodiazepines (BZD) only in alcohol detoxification in-patients (under clinical control). BZD treatment longer than 1 week is justified only when abstinence symptoms persist or in the case of an associated BDZ dependence. The French expert group recommended that, in no case should BZD treatment continue for more than 4 weeks. In agreement, the Socidrogalcohol guidelines (78, 79) recommend extreme caution in the use of BZD due to the greater risk of the development of a tolerance and dependence in patients with AUDs and AnxDs (77). Level of evidence: 2a.

Benzodiazepines should be used with caution because of the risk of abuse and overdose when associated with alcohol or other central nervous system depressants, such as opioids (45). Level of evidence: 2a.

Buspirone was found to be effective in comorbid AUD and AnxD patients (81, 82), an other clinical situations (83). In addition, buspirone was associated with greater retention, anxiety reduction, slower return to excessive drinking, and a smaller number of days of drinking. All of the treated patients also received weekly relapse prevention therapy (82). Level of evidence: 1a.

#### Use of Anticonvulsants

Gabapentin is effective in AnxD and AUD treatment (84). An RCT with high- (1,200 mg/day) and low- (900 mg/day) dose gabapentin- vs. lorazepam-treated AUD patients reported an improvement in anxiety symptoms in the lorazepam patients. The high-dose gabapentin patients had less alcohol craving, less anxiety and less daytime sedation (85). Level of evidence: 1a.

Other anticonvulsants have shown a capacity for anxiety symptom improvement in AUD patients. A randomized and open-label trial of 182 AUD patients (86) found that topiramate, at a mean dose of 200 mg/day, was better than naltrexone, at a mean dose of 50 mg/day, at reducing alcohol intake and craving and anxiety throughout the 6-month study period. Level of evidence: 1b.

Moreover, the molecule with the best evidence that also includes indications for GAD on the labeling is pregabalin (87). An RCT was published comparing pregabalin and naltrexone (88). The alcohol drinking index and craving scores were not significantly different between the groups. However, compared with naltrexone, pregabalin resulted in greater improvement in specific symptoms in the areas of anxiety, hostility, and psychosis, and survival function (duration of abstinence from alcohol). Level of evidence: 1a.

The mechanism involved in the efficacy of pregabalin in relapse prevention could be more associated with anxiety control than with craving (87, 89). Additionally, the available literature suggests important clinical abuse potential of pregabalin. Therefore, prescribers should pay attention to the signs of abuse (90, 91).

#### Use of Opioid Antagonists

An RCT comparing naltrexone vs. disulfiram was performed in AUD patients. The investigators found an improvement in anxiety symptoms in both naltrexone and disulfiram patients. There was no significant advantage of the combination of both medications (92). Level of evidence: 1a.

Moreover, there was a high rate of abstinence across groups. PTSD patients treated with an active medication (naltrexone, disulfiram, or in combination) had significantly better abstinence and craving results. There was also an overall improvement in the psychiatric symptoms of PTSD (93). Level of evidence: Ia. Curiously, the group treated with disulfiram also reported fewer PTSD symptoms. Here, abstinence could be the key factor in the improvement of the PTSD symptoms.

In contrast, Nalmefene, is an μ and δ opioid receptor antagonist and κ partial agonist. Nalmefene can be administered safely in alcohol dependence and can significantly prevent relapse to heavy drinking (94). Level of evidence: Ib. No studies involving this drug in comorbid AnxDs and AUDs have been published.

### Comorbid AnxD and AUD Psychotherapeutic Interventions Considerations

An RCT showed that combined therapy (medication + therapy) clearly reduced anxiety symptoms (95). It had no significant effect on alcohol relapse rates (95). Level of evidence: 1a.

A systematic review concluded that motivational interviewing and cognitive–behavioral interventions were associated with significant reductions in alcohol consumption and depressive and/or anxiety symptoms (96). Although brief interventions were associated with significant improvements in mental health and alcohol variables, longer interventions produced even better outcomes (72, 95–97). Level of evidence: 1a.

## Discussion

### General Considerations

There is insufficient evidence to confirm that the presence of comorbid AnxDs and AUDs has a negative effect on treatment results with regard to the manifestation of their separate forms (1, 15). The available literature is challenging to interpret because of the substantial heterogeneity of the studies with respect to study design, diagnostic assessment variability, type of treatment, and different clinical populations; all of these factors could explain the lack of relevant and conclusive information (15).

Regarding gender, various randomized clinical trials have examined whether AnxDs, when diagnosed at the beginning of the trial, were associated with alcohol consumption in women. RCTs in female AUD patients with AnxDs showed a weak association, with more days of drinking during the 6-month follow-up. Moreover, the presence of AnxDs during the trial was related to actual drinking (49). An early start in the treatment of AnxDs may be appropriate, as well as not waiting until alcohol problems are addressed, at least among those who have mild-to-moderate alcohol problems (47). Other authors recommend 2–4 weeks of abstinence before initiating treatment, using drugs with low abuse potentials, so the BZD should be avoided. The use of medication should be reserved for persistent anxiety, evaluating the risk of intoxication and interactions with alcohol or drug abuse (45, 78).

It would be useful to examine the potential risks of cross-recruitment for the different psychopharmacological categories, given that the psychiatric drugs may be affected by substance abuse and those patients can perhaps abuse the psychiatric drug itself. The clinic should carry out an evaluation of the possible interactions between the pharmacological treatment and the substances of abuse, as well as paying attention to the possible implications for the patient’s mental health.

The start of the psychopharmacological treatment in patients with comorbid AnxD and AUD requires a detailed clinical evaluation of the benefits/risks profile. The pharmacological interactions should be taken into account when choosing the psychotropics. Much care should also be taken with the SSRIs, as some symptoms of abstinence from alcohol can become superimposed or even added to the serotoninergic activation. For this reason, it is necessary for the clinic to pay careful attention to the interactions and to the precise identification of the symptoms so as to be able to control the effects and risks resulting from the medication itself (98, 99). The current tendency, however, is to recommend the use of the necessary medication while taking due precautions and with a high degree of care to identify any adverse effect derived from the medication or its interaction with alcohol or drug abuse, not to forget vigilance concerning the possible effects on the patient’s mental health.

#### Posttraumatic Stress Disorder

Posttraumatic stress disorder (PTSD) and SUD frequently co-occur, and their combination can increase poor health outcomes as well as mortality. The shared neurobiological features of these two illnesses include amygdalar hyperactivity with hippocampal, medial prefrontal, and anterior cingulate cortex dysfunction (100–102).

Regarding age, the findings contain preliminary evidence for the trans-diagnostic anxiety sensibility concept that encompasses a variety of anxiety manifestations (21). This condition is associated with greater posterior alcohol consumption problems. The relationship between the presence of a trauma (with or without PTSD) and AUDs has been reported (21, 80). From the data available, it could be deduced that later problems with AUD could be avoided if anxiety sensitivity in adolescents was treated first.

A systematic review of the Cochrane Database (1) showed the need for further research. Although there have been multiple RCTs for SSRIs, paroxetine and sertraline for PTSD above all, the evidence base for the effectiveness of medication in treating AnxDs and comorbid AUDs is currently inconclusive. The majority of the data for the efficacy and tolerability of medication were for SSRIs (paroxetine) in the case of social anxiety and AUDs (1).

Naltrexone, an opioid receptor antagonist with approved indications for alcohol relapse treatment in AUDs, has alleviated the severity of the PTSD symptoms in various open trials (86, 88, 92, 93). However, a later 12-week naltrexone and disulfiram RCT in veterans did not show that naltrexone medication was significantly more effective than placebo in the reduction of PTSD symptoms (92).

It seems that patients with PTSD should be examined carefully because this comorbidity is a major confound in terms of etiology, and there is not one treatment with clear evidence of efficacy in PTSD and AUD cases. The results for treatment medications remain inconclusive because of contradictory results. However, most studies have suggested a combination of interventions. Despite the contradictory results, it has been found that individuals with AUD and PTSD can safely be prescribed medications used in non co-occurring populations, and patients improve with treatment (101).

### Comorbid AD and AUD Pharmacological Treatment Considerations

#### Atypical Antipsychotics

A systematized review in non-comorbid conditions (60) supported the evidence that only a few atypical antipsychotics are effective in only a minority of the off-label conditions in which they are currently used, confirming that atypical antipsychotics are not all the same. Their use should be determined on the basis of a balance between efficacy and side effects, their characteristics and the patient’s preference.

#### Use of Antidepressants

Selective serotonin reuptake inhibitors are the first choice drugs in non-comorbid anxiety and AUD treatments (1, 15, 102). However, their effectiveness in AnxDs and AUDs is not so clear. There seems to be quite a large amount of data on the effectiveness of the SSRIs; however, there have been few rigorous studies, reflected in the very low quality of evidence that the results provide (1). High general levels of treatment discontinuation were observed in some of the randomized controlled trials. Consequently, there is little evidence of any effect of the SSRIs on abstinence from alcohol use, with the exception of the tricyclic antidepressant desipramine (1). However, at the clinical level, tricyclic antidepressants are not recommended due to their adverse effects and interactions (45).

The effectiveness for the SSRIs for alcohol consumption reduction is not very convincing. There is some evidence that the SSRIs can produce favorable results in the treatment of patients with less severe alcohol dependence, but these drugs can worsen the alcohol consumption results when combined with Cognitive–Behavioral Therapy in severe alcohol dependence patients (45). It should be emphasized that, in some trials, SSRIs were found to be inferior to placebo in the treatment of AUDs, demonstrating a sort of facilitating effect with regard to relapse. This point is relevant, and clinicians need to know it. Of course, when the comorbid disorder is relevant, there is probably a place for this treatment, but when the role of the AUD is prevalent, some reflections must be undertaken.

The reduction of social anxiety symptoms reported by patients (as part of an alcohol detoxification protocol) treated with mirtazapine and noradrenergic and specific serotonergic antidepressants suggested that drugs that target the neurotransmitter systems involved could be beneficial in the treatment of anxiety, as well as AUDs (45, 64). These results were later not confirmed in antidepressants such as venlafaxine, which act on both the serotonergic and noradrenergic systems.

#### Use of Anxiolytics

There are strong evidence and recommendations supporting the use of BZD for the treatment of the symptoms and signs of alcohol withdrawal syndrome and the prevention of seizures. Its continuous use is, however, not recommended (1, 45, 80). The use of BZD revealed no predictive effects of relapse or recovery in AUD patients in long-term treatment (81). Buspirone was found to be effective in comorbid AUD and AnxD patients (77, 81, 82). The use of BZD revealed no predictive effects of relapse or recovery in AUD patients in long-term treatment (1). The NMDA receptor antagonist-benzodiazepine has been involved in abuse liability (26, 102). So these medications should be used very carefully in the alcohol-dependent patients.

#### Use of Anticonvulsants

There have been promising results in the use of gabapentin and pregabalin in AnxD and AUD patients (87–92). However, there have been recent reviews of the possibility of pregabalin abuse and studies of cases concerning the abuse of gabapentin in patients with histories of substance abuse. As clinicians, we should be vigilant and remain alert to the appearance of signs of abuse among our patients (91).

#### Use of Opioid Antagonists

In AUD treatment, naltrexone injections did not provide any significant benefit in relapse, nor did it lower the numbers of binge drinks. Additionally, controlled clinical trials could not prove the efficacy of disulfiram, except in patients with a good adherence to treatment (1).

There is moderate evidence of efficacy for the off-label use of the drugs nalmefene and topiramate in the improvement of some alcohol consumption outcomes, and there is also limited evidence concerning valproic acid (1, 15, 45).

Acamprosate and naltrexone have the best evidence supporting their efficacy, but head-to-head trials have not consistently established the superiority of either medication. Thus, other factors can contribute to medication choices, such as frequency of administration, potential adverse events, and the availability of treatments.

### Comorbid AD and AUD Psychotherapeutic Interventions Considerations

Psychotherapeutic interventions have shown ample evidence of their efficacy in dual anxiety treatment. The NICE guidelines recommend addiction-focused counseling and training in coping strategies for substance-induced AnxDs. Specific psychological, motivational, and cognitive behavioral techniques/therapies should be applied in disorders that remain after abstinence has been established (103).

The use of specific pharmacological treatment of AUDs comorbid with AnxDs could be modulated on the basis of craving typologies. Identifying a craving type may represent an important predicting or matching variable for anti-craving psychotropics that could be determined using specific rating strategies (104).

Table 3 shows a summary of proposed recommendations for the treatment of patients with comorbid AnxDs and AUDs, based on the review of the literature performed.

**Table 3:** Recommendations for the use of psychopharmacological treatment in patients with comorbid AnxDs and AUDs.

Strength of recommendation^a^ (41)	Conclusions/level of evidence^b^ (46)
C	Low dosage of sedative antipsychotics for AnxD treatment in AUD patients
	Level of evidence: 4

C	Noradrenergic and specific serotonergic antidepressants: preliminary evidence was found: combined treatment—psychotherapy and mirtazapine—has an impact on the remission of AnxD symptoms in AUD patients
	Level of evidence: 4

A	Antidepressants: non-selective monoamine reuptake inhibitors—nefazodone has shown efficacy in anxiety symptom remission and in the reduction of number of drinks
	Level of evidence: 1a

A	Antidepressants: selective serotonin reuptake inhibitors. It was with paroxetine that the most scientific evidence was found for the AnxD treatment in AUD patients. In the same manners, various RCTs showed the efficacy of sertraline in PTSD treatment in AUD patients
	Level of evidence: 1a

B	Caution in the use of benzodiazepines (BZD) is recommended due to a greater risk of developing tolerance and dependence in patients with alcohol use and AnxDs
	Level of evidence: 2a

A	The use of BZD is recommended only for alcohol detoxification patients under clinical monitoring; in no case should the treatment be longer than 4 weeks
	Level of evidence: 1a

A	Buspirone was found effective for alcohol-dependent patients with comorbid anxiety
	Level of evidence: 1a

A	Gabapentin was found effective for anxiety and AUD treatment
	Level of evidence: 1a

A	Pregabalin was found effective in the remission of specific symptoms for anxiety, hostility and psychosis
	Level of evidence: 1a

A	Topiramate has shown the capacity to improve symptoms of anxiety in patients with alcohol use disorder
	Level of evidence: 1b

A	Use of opioid antagonists in patients with AnxD and AUD. Naltrexone was found to be effective in the remission of AnxD and improvement of abstinence rates
	Level of evidence: Ia.

A	Psychotherapeutics was found effective in the treatment of dual anxiety
	Level of evidence: 1a

*^a^The OCEBM (41) Grades of Recommendation: A, consistent level 1 studies; B, consistent level 2 or 3 studies or extrapolations from level 1 studies; C, level 4 studies or extrapolations from level 2 or 3 studies*.

*^b^The OCEBM (41) Levels of Evidence: 1a, systematic review (with homogeneity) of prospective cohort studies; 1b, prospective cohort study with good follow-up; 2a, systematic review (with homogeneity) of 2b and better studies; 4, case-series or superseded reference standards*.

*AnxD, anxiety disorder; AUD, alcohol use disorder*.

## Conclusion

That AUD and AnxD can reinforce one another (mutual-maintenance) indicates that treatments targeting both pathologies can be effective. In patients with mild and moderate AnxD with comorbid AUD, the treatment of the AnxD should commence as early as possible: this also applies to adolescent patients with simultaneous anxiety (with or without PTSD) and alcohol dependence. Women have higher levels of stress and AnxD, and they are more vulnerable to maintaining alcohol consumption levels: more intensive interventions should be considered for women with AnxD–AUD. Although the psychotherapeutic approach is effective for treating comorbid AnxD–AUD patients and the psychopharmacology approach should be done, there is, however, less evidence concerning the kind of drugs that have to be used.

Buspirone (77, 81, 82), gabapentine (84, 85), and pregabaline (87–89) have shown their efficacy in patients with comorbid AnxD–AUD, but potential abuse of gabapentine and pregabaline should be taken into account (90, 91). A study (86) shows that Topiramate could improve anxiety symptoms in alcohol-dependent patients, while some benefits with naltrexone have been described (92, 93). Paroxetine was found to be effective in social anxiety plus AUD (71–73) and Sertraline (74–76) is effective for PTSD among alcohol-dependent patients, especially in lower risk/severity of alcoholism. However, SSRIs should be used with caution when patients are actively drinking because they may increase alcohol consumption (78, 79).

According to these guidelines, all the patients should be prescribed, but with a high level of follow-up. Due to the lack of evidence, there is a need for more specific studies in this comorbid patient population, including the study of different types of patients and gender perspectives, which would shed light on drug therapies, as well as therapy combinations, treatment periods, and the existence of adaptations for clinical and special subpopulations.

## Author Contributions

CG and MD performed the literature review and wrote the preliminary version of the article. Analysis of the publications and clinical guides was undertaken by all the authors. MD, CR, and FJA conceived and designed the study. All the authors participated in the final writing of the article and approved the final version.

## Conflict of Interest Statement

CG has received fees to lecture for Janssen-Cilag, Servier, Lundbeck, Reckitt Benckiser Pharmaceuticals, and Lilly. The author has no other relevant affiliations or financial involvement with any organization or entity with a financial interest in or financial conflict with the subject matter or materials discussed in the manuscript, apart from those disclosed. MD has received fees to lecture over the last 4 years for the following entities: Pfizer and Adamed. She has received financial compensation for participation in the SASMAT-BUNHER project, which was funded by a grant from Reckitt Benckiser. The author has no other relevant affiliations or financial involvement with any organization or entity with a financial interest in or financial conflict with the subject matter or materials discussed in the manuscript, apart from those disclosed. CR has received fees to lecture for Janssen-Cilag, Bristol-Mayers Squibb, Ferrer-Brainfarma, Pfizer, Reckitt Benckiser/Indivior, Lundbeck, Otsuka, Servier, Lilly, GSK, Astra, Gilead, and MSD. He has received financial compensation for his participation as a member of the Janssen-Cilag, Indivior, Gilead, and MSD boards. He participated in the PROTEUS project, which was funded by a grant from Reckitt Benckisert. The author has no other relevant affiliations or financial involvement with any organization or entity with a financial interest in or financial conflict with the subject matter or materials discussed in the manuscript, apart from those disclosed. VB-M has received grants and has served as a consultant, advisor or Continuing Medical Education (CME) speaker over the last 4 years for the following entities: Angelini Spain; Angelini Portugal; Bristol-Myers-Squibb; Ferrer; Janssen; Juste; Lundbeck; Nutrición Médica; and Otsuka. PV has received grants and served as a consultant, advisor, or CME speaker over the last 4 years for the following entities: Janssen; Juste; Lundbeck; and Lilly. NS has received fees to lecture for Janssen, Lundbeck, Servier, and Lilly. He has received financial compensation for his participation as a member of the Janssen and Lundbec boards. The author has no other relevant affiliations or financial involvement with any organization or entity with a financial interest in or financial conflict with the subject matter or materials discussed in the manuscript, apart from those disclosed. FJA has received grants and served as a consultant, advisor, or CME speaker over the last 4 years for the following entities: Reckitt Benckiser, Indivior, and Shire.

## Abbreviations

AnxD, anxiety disorder; AUD, alcohol-use disorder; AWS, alcohol withdrawal syndrome; BAP, British Association of Psychopharmacology; BZD, benzodiazepines; CBT, Cognitive–Behavioral Therapy; CME, Continuing Medical Education; DSM, Diagnostic and Statistical Manual of Mental Disorders; DT, distress tolerance; EMCDDA, European Monitoring Center for Drugs and Drug Addiction; GABA, Gamma-aminobutyric acid; GAD, generalized anxiety disorder; GPR, Good Practice Recommendations; NaSSA, Noradrenergic and Specific Serotonergic Antidepressants; NMDA, *N*-methyl-d-aspartate receptor; NIDA, National Institute on Drug Abuse; OCD, Obsessive–Compulsive Disorder; OR, odds ratio; PTSD, posttraumatic stress disorder; RCT, randomized clinical trials; SEPD, spanish society on dual disorders; SNRIs, serotonin noradrenalin reuptake inhibitors; SSRI, selective serotonin reuptake inhibitor antidepressants; SMH, self-medication hypothesis; SUD, substance-use disorder; TRH, tension reduction hypothesis.
